# Inhibition of epidermal growth factor receptor signalling reduces hypercalcaemia induced by human lung squamous-cell carcinoma in athymic mice

**DOI:** 10.1038/sj.bjc.6603828

**Published:** 2007-05-29

**Authors:** G Lorch, J L Gilmore, P F Koltz, R M Gonterman, R Laughner, D A Lewis, R L Konger, K S Nadella, R E Toribio, T J Rosol, J Foley

**Affiliations:** 1 Department of Veterinary Biosciences, The Ohio State University, Columbus, OH 43210, USA; 2 Medical Sciences, Indiana University School of Medicine, Bloomington, IN 47405, USA; 3 Department of Dermatology, Indiana University School of Medicine, Indianapolis, IN 46202, USA; 4 Department of Biochemistry & Molecular Biology, Indianapolis University School of Medicine, Indianapolis, IN 46202, USA; 5 Department of Pathology and Laboratory Medicine, Indianapolis School of Medicine, Indianapolis, IN 46202, USA; 6 Department of Human Cancer Genetics, The Ohio State University, Columbus, OH 43210, USA

**Keywords:** PTHrP, anilinoquinazolines, lung cancer, hypercalcaemia, gefitinib, ZD1839

## Abstract

The purpose of this study was to evaluate the role of the epidermal growth factor receptor (EGFR) in parathyroid hormone-related protein (PTHrP) expression and humoral hypercalcaemia of malignancy (HHM), using two different human squamous-cell carcinoma (SCC) xenograft models. A randomised controlled study in which nude mice with RWGT2 and HARA xenografts received either placebo or gefitinib 200 mg kg^−1^ for 3 days after developing HHM. Effectiveness of therapy was evaluated by measuring plasma calcium and PTHrP, urine cyclic AMP/creatinine ratios, and tumour volumes. The study end point was at 78 h. The lung SCC lines, RWGT2 and HARA, expressed high levels of PTHrP mRNA as well as abundant EGFR protein, but very little erbB2 or erbB3. Both lines expressed high transcript levels for the EGFR ligand, amphiregulin (AREG), as well as, substantially lower levels of transforming growth factor-*α* (TGF-*α*), and heparin binding-epidermal growth factor (HB-EGF) mRNA. Parathyroid hormone-related protein gene expression in both lines was reduced 40–80% after treatment with 1 *μ*M of EGFR tyrosine kinase inhibitor PD153035 and precipitating antibodies to AREG. Gefitinib treatment of hypercalcaemic mice with RWGT2 and HARA xenografts resulted in a significant reduction of plasma total calcium concentrations by 78 h. Autocrine AREG stimulated the EGFR and increased PTHrP gene expression in the RWGT2 and HARA lung SCC lines. Inhibition of the EGFR pathway in two human SCC models of HHM by an anilinoquinazoline demonstrated that the EGFR tyrosine kinase is a potential target for antihypercalcaemic therapy.

Humoral hypercalcaemia of malignancy is a paraneoplastic disorder commonly associated with increased synthesis and secretion of parathyroid hormone-related protein (PTHrP) (Burtis *et al*, 1988). Studies from industrialised nations in the 1970s and 1980s, as well as more recent studies from other countries, report that patients with squamous-cell carcinoma (SCC) of the lung have the highest frequency of humoral hypercalcaemia of malignancy (HHM; ranging from 27 to 66%) as compared to other tumour types (Bender and Hansen, 1974; Mundy and Martin, 1982; Lazaretti-Castro *et al*, 1993). The precise mechanisms that activate high levels of PTHrP gene expression in tumours that are associated with HHM have yet to be identified. The diagnosis of HHM has a poor prognosis with a survival of less than 3 months (Wimalawansa, 1994). Hypercalcaemia has clinical effects on multiple organs such as fatigue, psychosis, confusion, vomiting, renal failure, constipation and cardiac arrest, which become life threatening as the syndrome progresses.

The ErbB pathway genes, especially the epidermal growth factor receptor (EGFR), are frequently overexpressed in SCC of the lung (Franklin *et al*, 2002). Lung SCCs produce a wide range of cytokines and growth factors, including those of the epidermal growth factor (EGF) family, such as transforming growth factor-*α* (TGF-*α*) and amphiregulin (AREG) (Rusch *et al*, 1993; Piyathilake *et al*, 2002). Activation of EGFR cytoplasmic receptor tyrosine kinases regulate essential cellular functions including proliferation, survival, migration, and differentiation, and play a central role in the genesis and progression of solid tumours (Jorissen *et al*, 2003). The prominent role of EGFR signalling in many tumour types has prompted the development of pharmacological inhibitors such as the anilinoquinazolines (gefitinib and erlotinib), which disrupt EGFR kinase activity by binding the ATP pocket within the catalytic kinase domain (Wakeling, 2002). Gefitinib induced substantial clinical responses and reduced tumour burden in ∼10% of patients with chemotherapy–refractory non-small cell lung cancers; however, the majority of these patients had EGFR mutations that constitutively activated the antiapoptotic protein AKT (Baselga, 2002; Fukuoka *et al*, 2003; Kris *et al*, 2003; Giaccone *et al*, 2004). Also, a phase III survival trial failed to verify the clinical benefit of gefitinib treatment for non-small cell lung cancer patients. In contrast, a phase III trial evaluating erlotinib efficacy revealed a significant overall improved survival rate for all non-small cell lung cancer patients (Tsao *et al*, 2005). In the future, EGFR-targeted therapeutics may be routinely used in the treatment of non-small cell lung cancers, including SCCs.

Parathyroid hormone-related protein is a gene product regulated by EGFR that influences the pathogenesis of lung SCCs. Previously, we have demonstrated that autocrine stimulation of the EGFR pathway is a major activator of PTHrP gene expression in keratinocytes, and disruption of the EGFR in keratinocytes with the EGFR-tyrosine kinase inhibitor (TKI), PD153035 (PD), reduced PTHrP mRNA up to 80% (Cho *et al*, 2004). The role of EGFR signalling in the stimulation of PTHrP expression and subsequent development of HHM in patients with SCC of the lung is unknown. Extensive work on several human cancer lines suggests that activation of the Ras-mitogen-activated protein kinase (Ras-MAPK) pathway provides a general mechanism for activation of PTHrP gene expression (Li and Drucker, 1994; Aklilu *et al*, 1997, 2000). The Ras-MAPK pathway is regarded as the prototypic second messenger cascade downstream of EGFR (Yarden, 2001). We hypothesised that EGFR-induced Ras-MAPK signalling accounts for high levels of PTHrP expression, and contributes to HHM caused by lung SCC and the administration of EGFR receptor TKIs would decrease total plasma calcium concentrations.

In this study, we report the expression of the ErbB family of receptors and the endogenous production of EGFR ligand mRNA by two human lung SCC cell lines, RWGT2 and HARA, that have been reported previously to produce hypercalcaemia in athymic mice (Guise *et al*, 1993; Iguchi *et al*, 1996). We present evidence that autocrine stimulation of the EGFR pathway is a major contributor to PTHrP gene expression in both lines grown *in vitro.* Next, we compared EGFR signalling and PTHrP production in hypercalcaemic mice with HARA and RWGT2 xenografts. Finally, we demonstrated that administration of gefitinib reduced hypercalcaemia and decreased PTHrP production in both xenograft models.

## MATERIALS AND METHODS

### Cell lines, kinase inhibitors and antibodies

RWGT2 cells were obtained from G Mundy and T Guise (University of Texas Health Science Center, San Antonio, TX, USA) and maintained in high glucose Dulbecco's modified Eagle's medium with GlutaMax™ (Gibco, Invitrogen, Carlsbad, CA, USA), supplemented with 10% heat-inactivated fetal bovine serum, and Normocin™ 100 *μ*g ml^−1^ (InvivoGen, San Diego, CA, USA). The HARA cell line was obtained from Dr Haruo Iguchi (Shikoku Cancer Center, Matsuyama, Japan). The original patient had an increased PTHrP concentration and hypercalcaemia, but no apparent bone metastases. HARA cells were maintained as described for the RWGT2 cells. Cells were passaged at 90% confluence. *In vitro* experiments were performed when both RWGT2 cells and HARA were ∼90% confluent. For the *in vitro* kinase inhibitor assays, the TKIs, PD (Calbiochem, San Diego, CA, USA) and gefitinib (gift from AstraZeneca, London, UK), as well as the MEK inhibitor PD98059 (Calbiochem) were dissolved in dimethylsulphoxide (DMSO), stored at −20°C at a stock concentration of 2 mM, and used at a final concentration of 1 *μ*M. The drugs were added to cells for 6 h and control cells were treated with media containing 0.01% DMSO (vehicle). For the ligand and EGFR blocking studies, the anti-human goat AREG antibody (R&D Systems, Minneapolis, MN, USA) was resuspended in sterile phosphate-buffered saline (PBS) to yield a final concentration of 100 *μ*g ml^−1^. Both the anti-human EGFR blocking monoclonal antibody 225 (Santa Cruz Biotechnology, Santa Cruz, CA, USA) and anti-AREG antibody were used at 10 *μ*g ml^−1^ and cells were treated overnight. For the animal studies, gefitinib was purchased from a national corporate pharmacy.

For immunoblot analysis and/or immunoprecipitations, the following antibodies were used: Phospho-EGF receptor-Tyr1068 catalogue no. 2234, EGFR no. 2232, erbB2 no. 2242, phospho-p44/42 mitogen-activated protein kinase (MAPK; Thr202/Tyr204) no. 9101, p44/42 MAPK no. 9102 (Cell Signaling, Beverly, MA, USA), 4G10 anti-phosphotyrosine no. 06–427 (Upstate USA, Charlottesville, VA, USA), erbB3 (sc-285), erbB4 (sc-283) (Santa Cruz Biotechnology) and anti-*β*-actin (Sigma, St Louis, MO, USA).

### Xenografts in nude (*Foxn1*^*nu*^) mice, biochemical analysis and gefitinib administration

All experimental procedures were approved by The Ohio State University Institutional Laboratory Animal Care and Use Committee. After baseline blood and urine samples were collected, mice were preconditioned to avidly consume peanut butter by several 0.25 g offerings before tumour development. Six- to eight-week-old weight-matched (24–26 g) male *Foxn1*^*nu*^ nude mice (Harlan, Indianapolis, IN, USA) were injected subcutaneously over the dorsal scapular area with 1 × 10^6^ RWGT2 or HARA cells. Subcutaneous tumours were observable approximately 7 days after injection.

Animals were monitored and weighed every other day and when any of the following conditions occurred: (1) tumours were greater than ⩾1 cm^3^; (2) cancer-induced loss of muscle mass; or (3) body weight decreased by ⩾5%, total calcium concentrations were measured to determine if the mice were hypercalcaemic. Blood collection was performed via puncture of the mandibular facial artery or vein with a 22-gauge needle and collected into a microtainer tube with lithium heparin (Becton Dickinson and Company, Franklin Lakes, NJ, USA). Plasma total calcium concentrations were measured in 10 *μ*l of heparinised plasma using the Vitros DT-60 II clinical chemistry analyser (Johnson & Johnson, Cornilla, GA, USA). Animals were considered hypercalcaemic when total plasma calcium concentrations were ⩾12 mg dl^−1^.

Once hypercalcaemia was confirmed, mice were randomly allocated to treatment with: (a) 200 mg kg^−1^ of gefitinib (tablet formulation; AstraZeneca, London, UK) in peanut butter daily for three consecutive days (0, 24 and 48 h) or; (b) 0.25 g peanut butter for three consecutive days (0, 24 and 48 h). They were randomised into two groups such that there was no difference in the mean plasma total calcium values between the two at the time treatment was initiated. During treatment, mice were placed in a cage devoid of mouse chow and bedding and observed until the mixture of drug and peanut butter or peanut butter alone had been completely consumed, approximately 2 min. Blood (75 *μ*l) was collected to measure plasma total calcium concentrations at 0, 6, 24, 52 and 78 h from treated and control mice. As additional controls, two groups of nontumour-bearing mice were treated as above in (a) and (b). Urine was collected following spontaneous voiding on wax paper. To calculate cyclic AMP (cAMP):creatinine ratios, urine samples (100 *μ*l) were collected from mice before xenografting for baseline analysis and at pretreatment and 6, 24, 52 and 78 h after treatment with gefitinib. Urine was acidified by adding 10 *μ*l of 6 N HCl and stored at −80°C until analysis. Tumour diameters were serially measured with digital calipers, and tumour volumes were calculated by the formula: volume=width^2^ × length/2. At the 78 h time point, mice were killed with 70% compressed CO_2_ gas and tumour tissue was collected, weighed, snap-frozen in liquid nitrogen, and stored at −80°C for protein analysis.

### Urine cAMP and creatinine assays

Urine samples were diluted 1 : 2000 in 0.1 N HCl and cAMP levels were measured using a commercial enzyme immunosorbent assay kit (Assay Designs, Ann Arbor, MI, USA). Urine cAMP was normalised to creatinine concentrations, which was measured using a commercial microplate Jaffè reaction kit (Quanti Chrom Creatinine Assay, BioAssay Systems, Hayward, CA, USA).

### Measurements of PTHrP concentrations

To determine the effects of gefitinib on plasma PTHrP concentrations, blood samples were collected via mandibular puncture into microtainer tubes with lithium heparin (Becton Dickinson and Company) on ice. A 200 *μ*l sample of plasma was obtained from individual mice for each time point. Plasma PTHrP was measured at baseline (i.e., before xenografting) (*n*=17 RWGT2 and *n*=10 HARA mice) and 6 and 78 h after the initial treatment with gefitinib or placebo. Biologically active plasma PTHrP (1–86) was measured using a commercially available two-site immunoradiometric assay (Diagnostic Systems Laboratories Inc., Webster, TX, USA). The limit for detection of the assay was 0.3 pM.

### RNA isolation and quantitative real-time reverse transcriptase PCR

Total RNA was prepared using the Mini RNA Isolation II kit (Zymo, Orange, CA, USA) according to the manufacturer's instructions. Reverse transcription and quantitative real-time PCR (QRT-PCR) were performed as detailed by Cho *et al* (2004) for all transcripts of PTHrP, AREG, TGF-*α* and HB-EGF. Data were normalised by use of the ratio of the target cDNA concentration to glyceraldehyde-3-phosphate dehydrogenase (GAPDH) to correct for differences in RNA quantity between samples. The results represented in the figures were derived from experiments where the cDNAs were prepared at the same time and then analysed by QRT-PCR performed on one plate.

### Immunoblot analysis

Immunoblotting for erbB and extracellular signal-regulated kinase (ERK) proteins was performed as in Gilmore and Riese (2004) and Foley *et al* (2000).

For measurement of phosphorylation of MAPK, RWGT2 cells were seeded at a density of 5 × 10^5^ cells/100-mm dish 24 h before treatment with PD. After treatment, cells were washed with ice-cold PBS, lysed with the protein extraction buffer as above for the tumour lysates, protein concentration determined, resolved by sodium dodecyl sulphate-polyacrylamide gel electrophoresis, transferred to nitrocellulose, and subjected to immunoblot analysis as described previously.

### Transient transfection

Transient transfection was performed using Lipofectamine and Plus reagent (Invitrogen, Carlsbad, CA, USA) according to the manufacturer's instructions. Transfections were normalised to total protein. Otherwise, details are similar to Cho *et al* (2004). Total protein was measured with the BCA protein assay reagent kit (Pierce Biotechnology, Rockford, IL, USA). Results were reported as relative luciferase units (RLUs), which represent normalised luciferase values for the Ras and Raf co-transfections divided by the normalised luciferase value of the empty vector co-transfections.

### Terminal deoxynucleotidyl transferase-mediated dUTP nick end labelling staining

Apoptosis was detected utilising the *In situ* Cell Death Fluorescein Detection Kit (Roche Diagnostics, Mannheim, Germany). Briefly, after deparaffinisation, rehydration and washing in 1 × PBS, sections were treated with terminal deoxynucleotidyl transferase (TdT) enzyme mixture, covered and incubated in a humidified slide chamber for 60 min at 37°C in the dark. After fixation, positive control slides were permeabilised with DNase I for 10 min at 20°C to induce DNA strand breaks, before labelling procedures. The negative control slides were incubated with label solution without terminal transferase. Finally, the slides were rinsed three times in PBS and analysed under a fluorescence microscope using an excitation wavelength of 488 nm.

### Statistics

Results were expressed as the mean±s.e.m. of triplicate or quadruplicate measures. Unless specifically indicated statistical comparisons were based on two-tailed analysis of the Student's *t*-test. A probability value of *P*<0.05 was considered to be significant. Analysis of variances (ANOVAs) with repeated measures were used to analyse the time differences between groups. Plots with the variable time (baseline, pretreatment, 6, 24, 52 and 78 h) in the *x* axis are reported to show the trends over time.

## RESULTS

### Stimulation of the EGFR activates PTHrP gene expression in two hypercalcaemia inducing SCC lines

**Figure 1:**
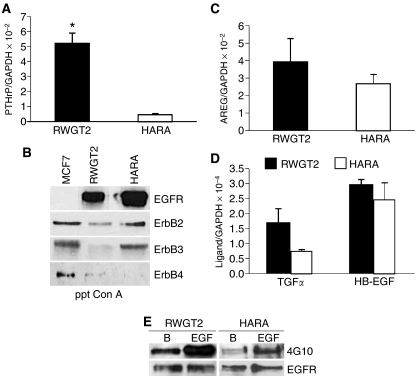
RWGT2 and HARA cells express predominately EGFR and ErbB2 receptors and produce EGF-related ligand mRNA for AREG, TGF-*α* and HB-EGF. (**A**) Parathyroid hormone-related protein/GAPDH mRNA ratios in RWGT2 and HARA cell cultures measured by QRT-PCR. The relative ratios of PTHrP mRNA to GAPDH mRNA levels were expressed as mean±s.e.m. of four cultures from a single experiment. ^*^P=0.003. (**B**) Relative epidermal growth factor family receptor expression as detected by western blots of cell extracts from RWGT2, HARA and the breast cancer cell line MCF-7. The right side of the figure indicates the specific erbB receptor antibodies. Protein extracts were precipitated using conA as indicated by ppt ConA on lower panel of the figure. (**C**, **D**) AREG, TGF-*α*, and HB-EGF/GAPDH mRNA ratios in RWGT2 and HARA cultures were measured by QRT-PCR. The relative ratios of EGF-like ligand mRNA to GAPDH mRNA level were expressed as mean±s.e.m. of four cultures from a single experiment. Note that the *y* axis for AREG mRNA is 100-fold greater than the *y* axis for TGF-*α* and HB-EGF mRNA. (**E**) Phosphorylation of the EGFR was measured in extracts from cells grown in basal medium. The band labelled with phosphotyrosine antibody reactivity was increased with EGF treatment (EGF). The general phosphotyrosine antibody 4G10 and EGFR antibodies used for protein detection are indicated on the right. These experiments were repeated four times with cells derived from independent passages with similar results.

The RWGT2 cell line was derived from a human lung SCC bone metastasis and the HARA cell line from a human primary lung SCC (Guise *et al*, 1993; Iguchi *et al*, 1996). At greater than 90% confluence, RWGT2 cells produce very high levels of all transcripts of PTHrP (close to 10^6^ copies per 2 *μ*g of cDNA) as detected by QRT-PCR (Figure 1A). In comparison, HARA cells at 90% confluence produced a PTHrP/GADPH mRNA ratio that was less than the RWGT2 cells (Figure 1A). As shown in Figure 1B, both cell lines expressed high levels of EGFR and moderate levels of the ErbB2 receptor as detected by Western blots of concanavalin-A (conA)-precipitated proteins. HARA cells expressed ErbB3 protein, whereas ErbB4 immunoreactivity was barely detectable in either cell line (Figure 1B). Both cell lines were evaluated for mRNA expression of the EGFR ligands; AREG, HB-EGF and TGF-*α* by QRT-PCR. The AREG/GAPDH mRNA ratios were two orders greater than HB-EGF and TGF-*α* in both lines (Figure 1C, D). To determine whether the EGFR was phosphorylated and active in the cells under normal basal culture conditions, Western blots of conA-precipitated protein extracts were probed with a general phosphotyrosine-specific antibody, 4G10. Extracts from the RWGT2 cells contained a phosphotyrosine immunoreactive band that increased in intensity in the control EGF-stimulated RWGT2 cells (left panel, Figure 1E). A small amount of phosphotyrosine immunoreactive EGFR was detected in HARA cells. Despite expressing similar levels of EGFR and EGFR ligand RNA, the HARA cells expressed less PTHrP mRNA *in vitro* than the RWGT2 cells.

**Figure 2:**
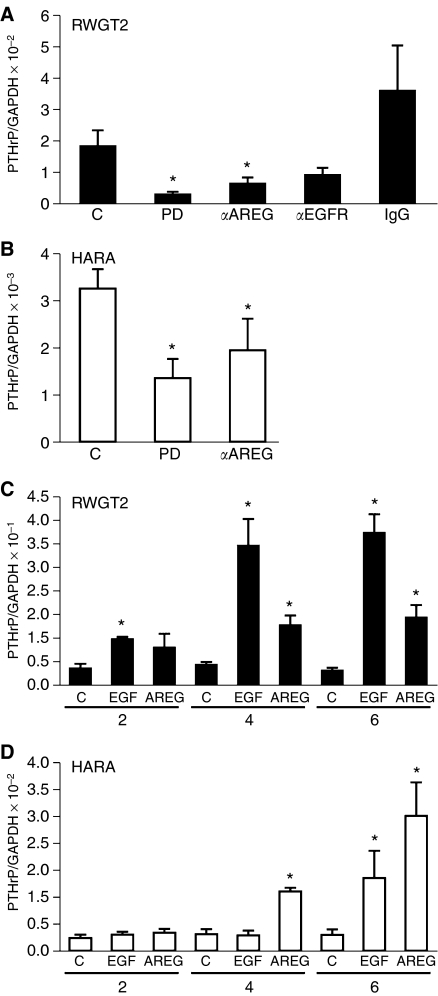
Parathyroid hormone-related protein mRNA expression is reduced by EGFR inhibitors and increased by the addition of exogenous EGFR ligands. (**A**) RWGT2 cells were treated with 1 *μ*M PD for 6 h, a neutralising polyclonal antibody to AREG (10 *μ*g ml^−1^), a ligand blocking EGFR antibody (*α*EGFR, 10 *μ*g ml^−1^) or PBS (C) for 24 h. Neutralising antibodies to growth factors are represented by the symbol (*α*). The mRNA was harvested from four independent cultures and analysed by QRT-PCR. PD, *P*=0.03; *α*AREG, *P*=0.05. (**B**) HARA cells were treated with 1 *μ*M PD for 6 h or a neutralising polyclonal antibody to AREG (10 *μ*g ml^−1^) or PBS (C) for 24 h. The mRNA was harvested from four independent cultures and analysed by QRT-PCR. PD, *P*=0.017; *α*AREG, *P*=0.034. (**C**) RWGT2 cells were incubated with PBS (C), 100 ng ml^−1^ of EGF or 1 mg ml^−1^ of AREG for 2, 4 and 6 h. The mRNA was harvested from four independent cultures and analysed by QRT-PCR. Two-hour EGF, *P*=0.02; 4 h EGF, *P*=0.002; 4 h AREG, *P*=0.002; 6 h EGF, *P*=0.004; 6 h AREG, *P*=0.001. (**D**) HARA cells were incubated with PBS (C), 100 ng ml^−1^ of EGF or 1 mg ml^−1^ of AREG for 2, 4 and 6 h. The mRNA was harvested from four independent cultures and analysed by QRT-PCR. Four-hour AREG, *P*=0.02; 6 h EGF, *P*=0.02; 6 h AREG *P*=0.013. Values in all panels represent the mean of four samples from individual cultures±s.e.m. These experiments were repeated three times with cells derived from independent passages with similar results, ^*^P<0.05 relative to C (control). Two-tailed Student's *t*-test.

The role of EGFR activation and autocrine ligand production on PTHrP gene expression was further investigated using EGFR TKIs, anti-EGFR and anti-AREG antibodies in both cell lines. Additional studies evaluated the effect of EGFR ligand treatment on PTHrP gene expression by QRT-PCR. Six-hour treatments with 1 *μ*M of the EGFR TKI, PD, decreased the ratio of PTHrP/GAPDH mRNA by ∼80% in RWGT2 cells grown in basal medium (Figure 2A). The addition of EGFR ligand blocking and AREG neutralising antibodies to basal culture medium reduced PTHrP/GAPDH mRNA levels 50–65% in RWGT2, respectively (Figure 2A). Neutralising HB-EGF and TGF-*α* antibodies (applied at concentrations up to 10-fold greater than the AREG antibodies) failed to reduce PTHrP mRNA levels in the RWGT2 line (data not shown). Treatment of HARA cultures with PD and AREG neutralising antibodies reduced PTHrP/GAPDH mRNA levels ∼60 and 40%, respectively (Figure 2B). Both cell lines were treated with the EGFR ligands, EGF (100 ng ml^−1^) or AREG (1 mg ml^−1^) for 2, 4 and 6 h and evaluated for PTHrP mRNA levels. Addition of exogenous ligands increased PTHrP transcript levels up to 10-fold by 6 h (Figure 2C, D). These data support the concept that the EGFR is a potent regulator of PTHrP gene expression in RWGT2 and HARA cell lines.

### The MAPK signalling pathway activates PTHrP gene expression downstream of the EGFR

**Figure 3:**
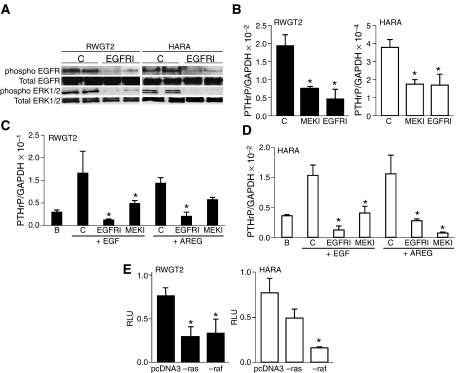
Mitogen-activated protein kinase signalling mediates EGFR-induced activation of PTHrP gene expression. (**A**) Epidermal growth factor receptor and ERK phosphorylation was reduced by EGFR TKI treatment in both RWGT2 and HARA cells. Upper bands are from a Western blot of conA-Sepharose precipitated proteins probed first with the generic phosphotyrosine monoclonal antibody 4G10, then stripped and reprobed with an EGFR antibody. Lower bands are from a Western blot of 30 *μ*g of protein probed first with a phospho-ERK 1/2 antibody, then stripped and reprobed with an antibody to ERK 1/2 and finally stripped and reprobed with an antibody to *β*-actin (not shown). C (control) indicates cells treated with DMSO vehicle for 6 h. EGFRI indicates cells treated with PD (1 *μ*M) for 6 h. This experiment was repeated twice. (**B**) A MEK inhibitor significantly reduced basal PTHrP/GAPDH mRNA ratios in both cell lines. RWGT2 and HARA cells were treated with 1 *μ*M DMSO (C), PD (EGFRI, 1 *μ*M) or PD98059 (MEKI, 10 *μ*M) for 6 h. The mRNA was harvested from four independent cultures and analysed. Two-tailed Student's *t*-test. ^*^P<0.05 relative to C (control). For the RWGT2 experiments: MEKI, *P*=0.045; EGFRI, *P*=0.03; for HARA experiments: MEKI, *P*=0.04; EGFRI, *P*=0.04. (**C**, **D**) A MEK inhibitor blunted EGFR ligand-induced increases in PTHrP/GAPDH mRNA ratios in both cell lines. Cells were preincubated with 1 *μ*M DMSO (C), PD (EGFRI, 1 *μ*M) or PD98059 (MEKI, 10 *μ*M) for 1 h and then stimulated with 100 ng ml^−1^ EGF (+EGF) or 1 *μ*g ml^−1^ AREG (+AREG) for 6 h. B represents untreated cells in all experiments. Panels B–D represent four replicates of samples with all experiments repeated twice. Two-tailed Student's *t*-test. ^*^P<0.05 relative to C (control). (**C**) EGF EGFRI, *P*=0.01; MEKI, *P*=0.05; AREG EGFRI, *P*=0.02; MEKI, *P*=0.053 not significant. (**D**) EGF EGFRI, *P*=0.001; MEKI, *P*=0.046; AREG EGFRI, *P*=0.049; MEKI, *P*=0.03. (**E**) Basal reporter gene activity from a PTHrP-P3 construct was reduced by a dominant-negative Raf construct in both the RWGT2 and HARA cells. RLU represents relative luciferase unit as defined in the Materials and methods. Co-transfection of the PTHrP reporter gene with the empty vector is indicated by pcDNA3, a dominant-negative Ras construct (−ras) a dominant-negative Raf construct (−raf). Values in all panels represent the mean of four samples from individual cultures±s.e.m. These experiments were repeated three times with cells derived from independent passages with similar results. Two-tailed Student's *t*-test. ^*^P<0.05 relative to C (control). RWGT2: ras, *P*=0.019; raf, *P*=0.033; HARA: ras *P*=0.02.

Several of the signalling pathways activated by EGFR converge on the MAPK cascade. Activation of the MAPK cascade was evaluated by examining the phosphorylation status of the downstream MAPK (ERK1/2) in basal culture conditions and after treatment with the EGFR TKI, PD. Six-hour treatments of both RWGT2 and HARA cells with 1 *μ*M PD resulted in a reduction of phosphorylated ERK1/2 as measured by Western blot without a change in the total ERK protein levels (Figure 3A). In addition, treatment with the MEK inhibitor, PD98059, reduced basal PTHrP mRNA levels in both lines to similar levels found in the EGFR TKI PD-treated cells (Figure 3B). Furthermore, treatment with the MEK inhibitor, PD98059, blunted the EGF- and AREG-stimulated increases in PTHrP gene expression in both lines (Figure 3C, D). Finally, co-transfection was used to test whether a dominant-negative Ras or Raf construct could disrupt basal expression from a human PTHrP-P3 promoter-driven reporter gene. As shown in Figure 3E, the dominant-negative Raf repressed reporter gene activity 50–70% in RWGT2 and HARA cells, respectively; however, the Ras construct only repressed reporter gene activity in the RWGT2 line. These results demonstrated that the effect of EGFR on PTHrP gene expression was mediated, in part, by the MAPK pathway in both hypercalcaemia-inducing lung SCC lines.

### Comparison of RWGT2 and HARA xenograft models of hypercalcaemia

**Figure 4:**
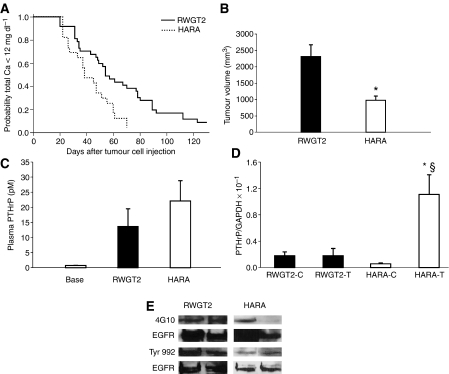
Comparison of RWGT2 and HARA models of hypercalcaemia. (**A**) Kaplan–Meir analysis of time to the development of hypercalcaemia. Fifty percent of mice developed HHM 40 days after injection of HARA cells compared to the RWGT2 xenograft mice that developed HHM 60 days after injection of cells. *P*=0.01. RWGT2 tumours (*n*=18) and HARA tumours (*n*=10). (**B**) Average tumour volume at time of hypercalcaemia. When hypercalcaemia was diagnosed, the RWGT2 (*n*=18) tumours were nearly twice as large as the HARA tumours (*n*=10) ^*^P=0.04 HARA compared to RWGT2. Two-tailed Student's *t*-test. (**C**) Comparison of plasma PTHrP concentrations in hypercalcaemic untreated mice with RWGT2 and HARA xenografts. Plasma PTHrP concentrations were determined 78 h after the development of HHM in both xenograft models. Average plasma PTHrP concentrations in the untreated mice were 80% higher (22 *vs* 12 pM) in the HARA (*n*=5) as compared to RWGT2-bearing mice (*n*=8). (**D**) Comparison of PTHrP mRNA expression between cells grown *in vitro* and *in vivo*. RNA was extracted from tumours that were removed 78 h after hypercalcaemia was identified. The ratio of PTHrP to GAPDH mRNA was assayed by QRT-PCR. The ratio of PTHrP to GAPDH mRNA in HARA tumours (HARA-T) was increased 100-fold compared to HARA cells (HARA-C) grown *in vitro*. The ratio of PTHrP to GAPDH mRNA in HARA tumours was sixfold higher than RWGT2 tumours (RWGT2-T). Values in all panels represent the mean of four samples from individual cultures or tumours±s.e.m. ^*^P=0.027 HARA tumours relative to HARA cells; ^§^*P*<0.05 HARA tumours relative to RWGT2 tumours. The QRT-PCR was repeated twice with similar results. Two-tailed Student's *t*-test. (**E**) Phosphorylation of the EGFR was measured in protein extracts from RWGT2 and HARA tumours by Western blotting. The general phosphotyrosine antibody 4G10 and polyclonal antibody for phosphorylated Tyr 992 was used to probe 1 mg of extracted tumour protein precipitated with conA-Sepharose. Blots were stripped and reprobed with EGFR antibodies. Four tumours from each cell line were evaluated.

The EGFR and MAPK pathway control PTHrP mRNA expression in both lines, however, there was a 10-fold greater level of PTHrP mRNA in the RWGT2 line as compared to HARA cells *in vitro*. To determine if both cell lines would be suitable for future studies with EGFR-targeted therapeutics, we compared the onset of hypercalcaemia, plasma PTHrP concentrations, tumour PTHrP mRNA expression and EGFR phosphorylation status in subcutaneous tumours of nude mice. The HARA line produced hypercalcaemia in less time after tumour cell injection than the RWGT2 cells (50% of HARA mice were hypercalcaemic in 40 days *vs* RWGT2 mice in which 50% of the mice were hypercalcaemic in 60 days) (Figure 4A). Moreover, the volume of the hypercalcaemia-inducing HARA tumours was ∼50% less than hypercalcaemia-inducing RWGT2 tumours (Figure 4B). Plasma PTHrP concentrations were ∼80%-fold higher (22 *vs* 12 pM) in mice with HARA as compared to RWGT2 xenografts. The RWGT2 cells grown *in vitro* or as xenografts, produced similar PTHrP mRNA levels (Figure 4C). In contrast, PTHrP mRNA levels were increased nearly 100-fold in the HARA xenografts compared to cells grown *in vitro* (Figure 4D). Tumour extracts from both lines had detectable phosphorylated EGFR immunoreactivity, but RWGT2 extracts produced more intense bands (Figure 4E). Therefore, both cell lines produced tumours with activated EGFR, secreted high concentrations of PTHrP, and induced hypercalcaemia.

### Gefitinib reduced hypercalcaemia in mice with RWGT2 and HARA xenografts

**Figure 5:**
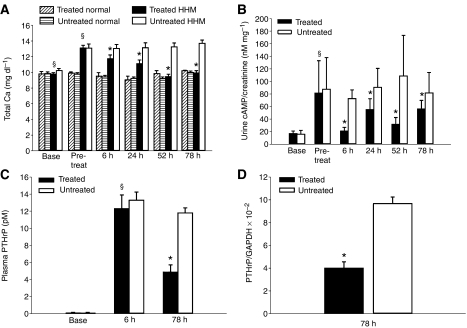
Gefitinib reduced plasma total calcium concentrations and urinary cAMP creatinine ratios in nude mice with RWGT2 xenografts and hypercalcaemia. (**A**) Hypercalcaemic mice with RWGT2 xenografts (*n*=9) were treated daily for 3 days with gefitinib (200 mg kg^−1^, per os (p.o.)). The first treatment occurred at the onset of hypercalcaemia. Treatment resulted in a statistically significant decrease in total plasma calcium concentrations compared to pretreatment values at all time points (6, 24, 52 and 78 h) and compared to untreated mice (*n*=8). Repeated measures one-way ANOVA, mean±s.e.m., ^*^P<0.001. Normal nude mice (*n*=5) were treated daily for 3 days with gefitinib (200 mg kg^−1^, p.o.) or untreated (*n*=5). Gefitinib did not decrease total plasma calcium concentrations compared to pretreatment values at all time points and compared to untreated mice. Repeated measures one-way ANOVA, mean±s.e.m. ^*^P=0.001 relative to treated; ^§^*P*=0.001 relative to baseline values. (**B**) Spontaneously voided urines were collected at the indicated times after the first gefitinib treatment (200 mg kg^−1^). Cyclic AMP and creatinine concentrations were measured. The cAMP and creatinine ratio was expressed as nM mg^−1^. Mice received additional doses of gefitinib at 24 and 48 h. Values at each time point represent the mean of two measurements from eight tumour-bearing mice from both the gefitinib-treated and untreated groups, as well as, 16 nontumour-bearing mice at baseline. Cyclic AMP levels were decreased in the gefitinib**-**treated mice when compared to untreated mice at all time points. Repeated measures one-way ANOVA, Mean±s.e.m., ^*^P=0.02 relative to treated; ^§^*P*=0.004 relative to baseline values. In all figures, the baseline and pretreatment groups are represented by the columns labelled base and pretreat, respectively. (**C**) Plasma PTHrP concentrations were measured using plasma samples collected before tumour injection (base) and at 6 and 78 h after gefitinib or placebo treatment. Experiments included nine gefitinib-treated mice and eight untreated mice. Baseline PTHrP values were from the same 17 mice. In the gefitinib-treated mice (black column), there was a mild reduction at 6 h followed by a marked reduction (50%) in plasma PTHrP concentrations when compared to untreated mice (white columns) at the respective time points. Untreated and gefitinib-treated mice with RWGT2 xenografts had PTHrP concentrations that were significantly greater than baseline at both 6 and 78 h. ^*^P=0.02 relative to treated; ^§^*P*=0.006 relative to baseline values. (**D**) Gefitinib treatment reduced PTHrP mRNA levels in RWGT2 tumours. RNA was extracted from tumours that were removed 78 h after hypercalcaemia was identified. The ratio of PTHrP to GAPDH mRNA was assayed by QRT-PCR. The ratio of PTHrP to GAPDH mRNA in the gefitinib-treated tumours (*n*=6) was decreased 60% as compared to untreated (*n*=6). The QRT-PCR was repeated twice with similar results. *P=*0.014.

The anilinoquinazolines, gefitinib, is a highly specific EGFR TKI that can be used in rodents and humans at high doses with minimal toxicity (Janmaat and Giaccone, 2003). Treatment with gefitinib (1 *μ*M) was as effective as PD (1 *μ*M) in reducing PTHrP mRNA and EGFR/ERK phosphorylation in RWGT2 cells (data not shown). To assess the ability of an anilinoquinazoline to decrease plasma total calcium concentrations *in vivo*, RWGT2 tumour-bearing mice with HHM were administered gefitinib (200 mg kg^−1^) every 24 h for three treatments and plasma total calcium concentration was measured at 6, 24, 52 and 78 h after the first treatment. The total calcium concentrations for normal 6- to 8-week-old male nude mice ranged from 8 to 10 mg dl^−1^ (Figure 5A). Total calcium concentrations of gefitinib-treated mice were significantly reduced at all time points when compared to pretreatment values and untreated mice at comparable time points (Figure 5A). The plasma total calcium concentrations of all gefitinib-treated mice returned to baseline at 52 h (*P*=0.001). Moreover, when normocalcaemic nude mice without tumours were given gefitinib (200 mg kg^−1^) on an identical dosage schedule, treatment did not reduce plasma calcium concentrations as compared to pretreatment and baseline values (Figure 5A).

Stimulation of the PTH receptor by PTHrP activates adenylyl cyclase in the renal tubules so we measured the effect of gefitinib administration on urinary cAMP:creatinine ratios. Gefitinib-treated mice with RWGT2 and HHM had a significant reduction of urine cAMP levels at 6, 24, 52 and 78 h when compared to the control mice (*P*=0.02) (Figure 5B).

The *in vivo* response of PTHrP gene expression to treatment with an EGFR TKI was evaluated by measuring plasma PTHrP concentrations and PTHrP mRNA from tumours in hypercalcaemic mice with RWGT2 xenografts. Plasma samples were collected before xenografting, 6 h after the first gefitinib treatment (6 h) as well as at the end of the trial, that is, 30 h after the third and final drug administration (78 h). Comparison of gefitinib- and vehicle-treated groups revealed a trend towards decreased PTHrP concentrations as early as 6 h after the first treatment. There were reduced PTHrP concentrations (*P=*0.01) in the gefitinib-treated mice at 78 h, but the PTHrP concentrations were not decreased to baseline values (Figure 5C). PTHrP/GAPDH mRNA ratios were reduced ∼60% in tumours from treated mice at 78 h (Figure 5D). These data demonstrated that gefitinib reduced PTHrP gene expression and circulating PTHrP concentrations in the RWGT2 model of HHM.

**Figure 6:**
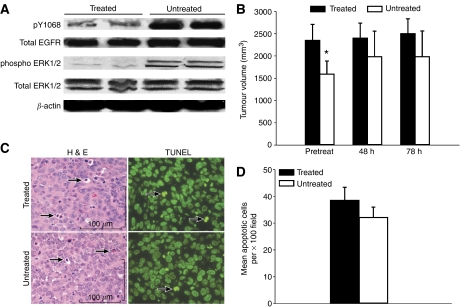
Gefitinib treatment reduced RWGT2 tumour EGFR and MAPK phosphorylation at 78 h but not tumour volume. (**A**) Comparison of EGFR and MAPK phosphorylation as assessed in untreated and gefitinib-treated RWGT2 tumours by Western blots. Tumours from untreated RWGT2 mice had greater phosphorylation of both EGFR phosphotyrosine residue 1068 and ERK1/2 when compared to tumour lysates from RWGT2 gefitinib-treated mice. Furthermore, all lysates had equivalent EGFR and ERK1/2 expression. Equal amounts of total protein (10 *μ*g) were separated by electrophoresis using 4–20% tris-glycine sodium dodecyl sulphate-polyacrylamide gels, transferred onto nitrocellulose membranes, and analysed by immunoblotting using rabbit polyclonal antibodies to phosphotyrosine 1068 and phospho ERK1/2. Blots were stripped and reprobed with rabbit polyclonal antibodies against EGFR and ERK1/2. The blots were stripped a final time and reprobed with a polyclonal antibody to *β*-actin. Experiments were repeated three times, and the data from a representative blot are shown. (**B**) Tumour volumes were measured serially as described in ‘Materials and methods.’ No reduction in tumour volume was present (pretreatment, 48 and 78 h) between gefitinib-treated and untreated mice at any time point. Each bar represents the mean tumour volume of eight or nine mice; bars, s.e.m. ^*^P=0.048 *vs* treated at the pretreatment time point even though the animals were randomised. (**C**) Gefitinib treatment of mice with RWGT2 xenografts did not cause an increase in apoptosis. Representative samples at 78 h from gefitinib-treated and untreated tumours. H&E staining revealed similar numbers of apoptotic cells represented as shrunken cells with eosinophilic cytoplasm and karyorrhectic or pyknotic nuclei in both the treated and untreated mice (black, arrows); bar 100 *μ*m. Fluorescent TUNEL staining for apoptosis (black, arrows) revealed similar numbers of apoptotic cells throughout the tumour parenchyma. × 400. (**D**) Comparison of numbers of apoptotic cells in gefitinib-treated and untreated RWGT2 tumours at 78 h. Ten × 100 fields from a fluorescent TUNEL slide for each mouse were counted to assess the number of apoptotic cells per field. The mean apoptotic cell number per × 100 field was represented as the black bar for gefitinib-treated and white bar for the untreated. No significant differences in mean apoptotic cells in tumours were present between the gefitinib-treated *vs* untreated mice.

Epidermal growth factor receptor phosphorylation in tumours was measured by Western blots with the EGFR phosphotyrosine-specific antibody to residue 1068. In RWGT2 tumours harvested at 78 h from treated mice, Y1068 phosphorylation was markedly reduced compared to the RWGT2 tumour lysates from untreated mice (Figure 6A). Phospho-ERK levels were decreased in the tumours from treated mice as well. Differences in the tumour volumes at the pretreatment time point were found between the treated and untreated groups; however, there was no difference in the tumour volume between these groups of mice at the 48 or 78 h time points (Figure 6B). No difference in final tumour weights between treated and untreated mice were found (data not shown). Degree of tumour cell apoptosis was measured with both TdT-mediated dUTP nick end labelling (TUNEL) and haematoxylin and eosin (H&E) at the 78 h time point in both the treated and untreated mice (Figure 6C). There were no differences in TUNEL labelling indexes or apoptosis (H&E) of tumour cells in the treated mice compared to the untreated mice (Figure 6D). The gefitinib-mediated reduction in PTHrP mRNA and plasma concentrations was not accompanied by a decrease in tumour volume or an increase in tumour cell apoptosis.

**Figure 7:**
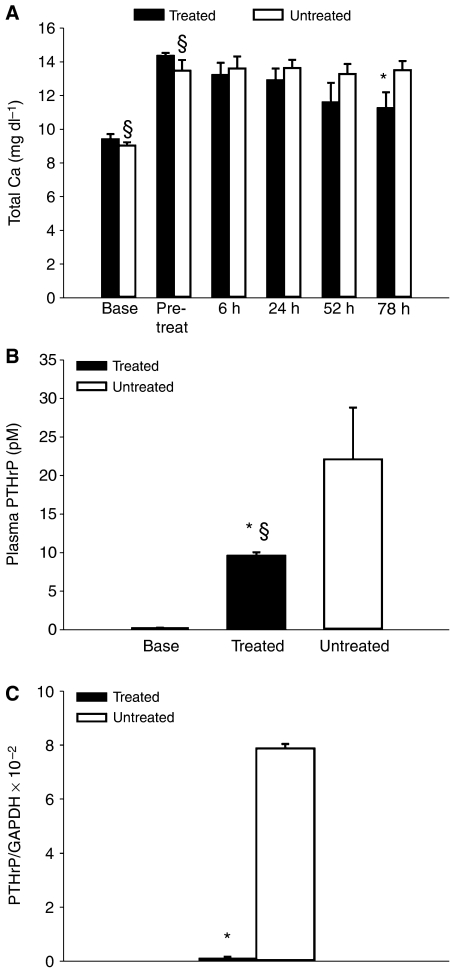
Gefitinib reduced plasma total calcium and PTHrP concentrations in nude mice with HARA xenografts and hypercalcaemia. (**A**) Hypercalcaemic mice with HARA xenografts (*n*=5) were treated daily for 3 days with gefitinib (200 mg kg^−1^, p.o.). The first treatment occurred at the onset of hypercalcaemia. Treatment resulted in a statistically significant decrease in total plasma calcium concentrations compared to pretreatment values at 78 h and compared to untreated mice (*n*=5). Repeated measures one-way ANOVA, mean±s.e.m., ^*^P=0.042 relative to treated; ^§^*P*=0.001 relative to baseline. (**B**) Plasma PTHrP concentrations were measured using plasma samples collected before HARA cell injections (baseline) and at 78 h after gefitinib or placebo treatment. Experiments included five gefitinib-treated mice and five untreated mice. Baseline PTHrP values were from the same 10 mice. In the gefitinib-treated mice (black column), there was a marked reduction (60%) in plasma PTHrP concentrations when compared to untreated mice (white columns). Untreated and gefitinib-treated mice with HARA xenografts had PTHrP concentrations that were significantly greater than baseline at 78 h. ^*^P=0.047 relative to treated; ^§^*P*=0.001 relative to baseline. (**C**) Gefitinib treatment reduced PTHrP mRNA levels in RWGT2 tumours. RNA was extracted from tumours that were removed 78 h after hypercalcaemia was identified. cDNA was produced with the MultiScribe Reverse Transcriptase Kit (Applied Biosystems, Foster City, CA, USA) and QRT-PCR performed. PTHrP to GAPDH mRNA was assayed by QRT-PCR. The ratio of PTHrP to GAPDH mRNA in the gefitinib-treated tumours (*n*=3) was decreased 90% as compared to untreated (*n*=4). The QRT-PCR was repeated twice with similar results. ^*^P=0.02.

In June of 2005, use of gefitinib in humans was restricted to lung cancer patients for compassionate use only and could not be purchased for research purposes. At this time, we possessed a small amount of gefitinib sufficient to treat five animals. This material was used to treat five hypercalcaemic HARA xenografts using a dose and time course identical to that we used with the RWGT2 cells. Total calcium concentrations were significantly reduced by 78 h (Figure 7A) (*P*=0.042). Urinary cAMP creatinine ratio averages were not significantly reduced at any time point (data not shown). At 78 h plasma PTHrP levels were significantly reduced compared to untreated mice, but were not reduced to the levels observed in treated mice with RWGT2 tumours (*P*=0.047) (compare Figure 5C to Figure 7B). HARA tumour PTHrP mRNA levels were decreased by 90% in the gefitinib-treated animals (Figure 7C).

## DISCUSSION

It has been known for over a decade that exogenous EGF stimulates PTHrP gene expression in a human lung SCC and keratinocyte lines (Tait *et al*, 1994; Heath *et al*, 1995), but it has not been determined whether EGFR signalling contributes to cancer-mediated syndromes such as HHM. We have established that the EGFR ligand, AREG, stimulated EGFR signalling and PTHrP gene expression in two human lung SCC lines that induce hypercalcaemia when grown *in vivo*. As with most lung SCCs (Salomon *et al*, 1995; Scagliotti *et al*, 2004; Prudkin and Wistuba, 2006), both HARA and RWGT2 cells expressed abundant EGFR *in vitro* and *in vivo*. Phosphotyrosine immunoblotting revealed that the receptor-associated kinase was active in both lines under basal conditions. Measurements of mRNA expression of the EGF family of ligands showed that both cell lines expressed high levels of AREG and modest amounts of HB-EGF and TGF-*α*. The coexpression of two or more EGF-like growth factors frequently occurs in human carcinomas (Ciardiello *et al*, 1991; Qi *et al*, 1994; Normanno *et al*, 2001). The ability of neutralising antibodies to AREG but not to TGF-*α* or HB-EGF to reduce PTHrP transcripts in both lines demonstrated that AREG was the primary activator of EGFR in cell lines. In both cell lines, exogenous EGFR ligands stimulated a four- to ninefold increase in PTHrP mRNA. Epidermal growth factor receptor signalling was necessary and sufficient for increased PTHrP gene expression in the HARA and RWGT2 cells. Whether signalling by the EGFR activates PTHrP gene expression by increasing gene transcription or message stability will be addressed by future studies.

The MAPK pathway mediated the EGFR-induced activation in the RWGT2 and HARA cells. Our findings are consistent with studies that reported calcium receptor (CaR) stimulation induced EGFR-MAPK signalling in transfected human embryonic kidney cells, human prostate cancer cells and hypercalcaemia-inducing rat Leydig cells (H-500) (MacLeod *et al*, 2003; Yano *et al*, 2004; Tfelt-Hansen *et al*, 2005b). Blockade of the Ras-MAPK pathway with a dominant-negative Raf construct or a MEK inhibitor (PD98059) reduced PTHrP gene expression in H-500 cells (Aklilu *et al*, 2000). A ras inhibitor, B-1086, was able to slow the growth of the H-500 tumour and prevented the formation of hypercalcaemia *in vivo* (Aklilu *et al*, 1997, 2000). Highly specific and low toxicity inhibitors of the MAPK pathway will likely prove useful to reduce PTHrP gene expression in certain cancers.

Investigations on the control of PTHrP gene expression suggest that different signalling events were responsible for the induction of hypercalcaemia in the RWGT2 and HARA models. Previous studies suggested that PTHrP gene expression *in vitro* predicted the ability of a cell line to induce hypercalcaemia in nude mice (Wysolmerski *et al*, 1996). The RWGT2 line was part of that study and consistent with those earlier findings we determined that the PTHrP mRNA levels *in vivo* were approximately equivalent to those in the tumour (Figure 4). In contrast, HARA tumours taken from hypercalcaemic mice demonstrated a ∼100-fold increase in PTHrP mRNA levels over cells *in vitro*, indicating that host factors activated PTHrP gene expression. Activation of PTHrP gene expression *in vivo* has been attributed to circulating factors as well as tumour microenvironment-derived cytokines or growth factors. Observations based on several xenograft HHM models suggest that high plasma calcium concentrations result in CaR-mediated activation of PTHrP production (Sanders *et al*, 2001; Tfelt-Hansen *et al*, 2003, 2005a). In the bone microenvironment, TGF-*β* released from bone at sites of osteoclastic resorption activates PTHrP expression in metastatic cancer cells (Kakonen *et al*, 2002). Tumour-associated stroma in soft tissues also produces TGF-*β* (Gatza *et al*, 2003; Trapani, 2005). Therefore, hypercalcaemia induced by the RWGT2 model appeals to be due to an intrinsic capacity of the cancer cells to produce PTHrP, whereas the HARA line induces the syndrome as a result of extrinsic stimulation of tumour cell PTHrP gene expression.

Epidermal growth factor receptor signalling is likely to play different roles in the control of PTHrP gene expression and generation of hypercalcaemia by the two cell lines. RWGT2 cells *in vitro* or in tumours exhibited relatively high levels of EGFR phosphorylation (Figures 1, 4). Consistent with a central role of the EGFR in control of PTHrP gene expression in RWGT2 cells, EGFR TKIs substantially reduced PTHrP *in vitro* as well as plasma protein levels, and this was accompanied by a rapid decrease in serum calcium levels (Figures 2 and 5). In contrast, EGFR phosphorylation was barely detectable in HARA tumours, suggesting that the receptor was not active to the extent observed in the RWGT2 line. The best understood mechanism of EGFR activation involves the autocrine/paracrine release of the ectodomain of integral membrane EGFR ligand proteins (Jorissen *et al*, 2003). The shedding of the ectodomain involves activation of matrix metalloproteinases and extracellular release of EGFR ligands (Yan *et al*, 2002). G-protein-coupled receptors such as the CaR, are able to transactivate EGFR through induction of the membrane-associated matrix metalloproteinases such as ADAM-17 (Ruhe *et al*, 2006). We have recently cloned and sequenced the CaR in both the HARA and RWGT2 cells lines (data not shown), but receptor characteristics typical of neoplastic cells such as overexpression, constitutive activation and the presence of activating mutations have not been evaluated. Although it is possible that signals from the CaR or other G-protein receptors might result in high levels of EGFR activity in the RWGT2 line, the lack of robust EGFR phosphorylation in the HARA tumours suggested transactivation did not account for increased PTHrP gene expression in this model. This does not eliminate the possibility that second messenger pathways directly coupled to the CaR could underlie the activation of PTHrP expression in the HARA line. The fact that gefitinib induced a decrease in the plasma calcium levels in the HARA model suggests a role for the receptor in activation of PTHrP gene expression in these cells. We speculate that this might reflect a synergism of EGFR signalling with another growth factor pathway such as TGF-*β*. In conclusion, autocrine AREG stimulation of the EGFR may serve as the intrinsic factor that drives high levels of PTHrP gene expression in the RWGT2 line, whereas this pathway appears to play a secondary role in the extrinsic activation of the gene in HARA tumours by host factors.

The effect of gefitinib in the RWGT2 model of HHM was due in part to direct actions on tumour cells. Gefitinib treatment reduced EGFR phosphorylation at tyrosine 1068 and ERK phosphorylation in the RWGT2 tumours consistent with a blockade of EGFR kinase activity. Tumour volume, mass and cell death were not altered in the treated mice indicating that apoptosis of the tumour cells did not contribute to the lower PTHrP concentrations. We speculate that blockade of EGFR-mediated activation of PTHrP gene expression contributed to the decreased plasma protein concentrations in both the RWGT2 and HARA models. However, over 20 nonspecific cellular targets of gefitinib unrelated to EGFR inhibition has been identified recently in HeLa cells using proteomics. Therefore, we are unable to rule out the possible contribution of additional kinase inhibition in decreasing hypercalcaemia in our *in vitro* studies (Brehmer *et al*, 2005).

Understanding the signalling events that regulate bone resorption has led to the development of a new generation of potential therapies for HHM. These include the soluble decoy receptor for RANKL, osteoprotegerin (OPG), anti-PTHrP antibodies and bisphosphonates. A single injection of anti-PTHrP antibodies reduced calcium and cAMP/creatinine levels near baseline levels within hours after administration in a mouse xenograft model of HHM (Kukreja *et al*, 1988) and humanised anti-PTHrP antibodies had a similar efficacy in a nude rat xenograft model of HHM (Sato *et al*, 2003; Onuma *et al*, 2005). One treatment with OPG reduced plasma calcium concentrations to baseline within 24–48 h in a mouse xenograft model of HHM (Morony *et al*, 1999). In the above models of HHM, treatment with the bisphosphonates, pamidronate, zoledronic acid and alendronate were unable to return serum calcium concentrations to baseline (Morony *et al*, 1999; Onuma *et al*, 2005).

In summary, our findings support the concept that EGFR signalling contributes to high levels of PTHrP gene expression in lung SCCs that induce HHM. Furthermore, EGFR TKIs have the capacity to reduce hypercalcaemia in two lung SCC models, and their use to treat various types of non-small cell lung cancer may prevent the development of HHM.
